# The Characteristic of Insect Oil for a Potential Component of Oleogel and Its Application as a Solid Fat Replacer in Cookies

**DOI:** 10.3390/gels8060355

**Published:** 2022-06-06

**Authors:** Doyoung Kim, Imkyung Oh

**Affiliations:** Department of Food Science & Technology, Sunchon National University, Suncheon 57922, Korea; kodosun@naver.com

**Keywords:** *Tenebrio molitor* larvae, insect oil, oleogel, fat replacer, cookie quality

## Abstract

The larvae of *Tenebrio molitor*, an edible insect, have recently attracted attention in the food industry as a protein supplement or future food material. However, despite more than 30% of the total weight being fat content, few studies have been conducted on the fat (oil) derived from *Tenebrio molitor* larvae (TM oil) and its food utilization. In this study, TM oil was extracted and its fatty acid composition and antioxidant activity were investigated. Then, the oleogels were prepared with TM oil and oleogelators (candelilla wax, carnauba wax, and beeswax) and their rheological and thermal properties were evaluated to elucidate their utilization as a solid fat replacer in cookies. In the results, TM oil contained 73.6% unsaturated fatty acids and showed a lower antioxidant activity than olive oil. Although the highest hardness was shown in oleogel with candelilla wax, the highest viscoelasticity above 50 °C was observed for oleogel with carnauba wax. The highest melting point was observed in carnauba oleogel. Lower peroxide values were observed in the oleogel samples than for TM oil, indicating that oleogelation of structuring oil improved the oxidative stability of TM oil. In addition, the shortening replacement with carnauba wax oleogel showed a desirable cookie quality in terms of spreadability and texture properties.

## 1. Introduction

With interest growing in edible insects, the market size is expected to exceed USD 112 million by the year 2023 [1]. More than 1900 edible insects have traditionally been consumed in various countries, including in Africa, Asia, Central America, and Europe [2], and insects have recently been recognized as a valuable protein source and a novel future food. This is because edible insects have an excellent production efficiency and lower carbon consumption than conventional protein sources such as meat, fish, and milk [3]. An increased human population requires more food consumption, especially protein sources, which have increased carbon consumption and contribute to climate change and environmental degradation. To address these challenges, edible insects have emerged as alternatives to reduce carbon consumption and provide nutrients to humans. However, negative perceptions of edible insects, such as off-flavor and disgust, have led to a lack of exploitation of this market, even though edible insects are beginning to be recognized as a food with high nutritional value [4]. Therefore, processing methods have also been developed to alleviate negative consumer perceptions and incorporate food materials. Insect powder (flour) made by drying and grinding processes can be produced in an unrecognizable form (powdered form) and applied to bakery products [5,6], snacks [7], and meat products [8]. Kroncke et al. [9] compared the drying processes (freeze-drying, fluidized-bed-drying, microwave-drying, and vacuum-drying) of *Tenebrio molitor* on protein, fat, and fiber contents, and Fombong et al. [10] also reported the influence of freeze-drying and oven-drying of *Ruspolia differens* on nutrient composition. Lipid and protein extraction methods are commonly used for insect processing. Insect proteins and lipids can be extracted using water, organic solvents, and enzymes to facilitate industrial processes and overcome the problems of insects. However, most studies have focused on the extraction efficiency and physicochemical properties of insect proteins, and few studies have been conducted on the use of insect fat (or oil) in foodstuffs. In South Korea, nine types of insects are approved as food: *Oxya japonica Thungberg*, *Bombyx mori*, *Batryticatus bombyx*, *Tenebrio molitor*, *Protaetia brevitarsis seulensis*, *Allomyrina dichotoma*, *Gryllus bimaculatus*, *Zophobas atratus*, and *Apis mellifera*. Among them, *Tenebrio molitor* belongs to the coleopteran family Tenebrionidae, which are famous as grain pests and are used as food for small animals such as birds and hedgehogs. Generally, the lipid content of insects ranges from 10% to 30% of their total weight and is relatively high in unsaturated fatty acids [11]. In accordance with the results of Tzompa-Sosa et al. [12], *Tenebrio molitor* contains 13% lipids and 75% unsaturated fatty acids, which are composed of oleic acid (C18:1) (49.5%) and linoleic acid (C18:2) (21.8%). As *Tenebrio molitor* oil (TM oil) is known to be a good source of unsaturated fatty acids that have a positive effect on human health, research is needed to utilize these functionalities for practical applications in various foods.

On the other hand, a novel technology for oleogelation, in which liquid unsaturated fat is converted into solid fat without chemical composition changes, has attracted attention. The oleogel is usually manufactured to structure liquid oil assisted with oleogelators, generating solid-like properties or three-dimensional structures. These oleogels have great potential as solid fat substitutes in various food models [13]. The solid fat (shortening, margarine, and butter) allows not only food functionality by imparting tenderness, mouthfeel, and flavor but also structure by aeration in the bakery model. They are easily handled in the baking process and have a higher oxidative stability compared to liquid vegetable oil. The solid fat provides many functions and properties due to saturated fatty acid, which is formed of a crystal network and structure-keeping liquid oil of fat [14]. Moreover, the oleogelation is described as structured solid-like materials with the entrapment of a high amount of liquid oil within a three-dimensional gel network. Due to this structural similarity, the oleogel has been recognized and used as a solid fat replacer in many studies. The solid fat can cause health problems including cardiovascular disease and metabolic syndrome, and it is desirable to use the oleogel as a solid fat replacer, which contains unsaturated fatty acid but has a solid form resulting in improving processability.

The functional and physicochemical properties of oleogels could be affected by the gelator type (vegetable waxes, monodiglycerides, alcohols or esters of fatty acids, phospholipids, and phytosterols), fatty acid composition, or chain length [15,16]. Thus, the selection of oil type or oleogelator is important for developing new oleogels. However, to the best of our knowledge, no research has been conducted on the preparation of oleogels containing edible insect oils and on the physicochemical properties of insect oil-oleogel depending on oleogelator types. Natural wax, one of oleogelators, is obtained from plant sources and frequently applied to prepare the oleogel for food products because of its safety approval, cost-effectiveness, and utilization [17]. Moreover, the strong oleogelation ability of natural wax is the one of reasons it is widely used in food, cosmetics, and pharmaceuticals. Each natural wax has different melting points and crystallization behaviors, indicating the different rheological and chemical properties [18,19]. For this reason, the research was conducted to compare the utilization of wax-based oleogel as a solid fat replacer depending on the gelator type in the food model. Oh et al. [18] reported that the replacement of shortening with beeswax/sunflower oil-oleogel could produce a cake characterized by a similar specific volume and lower saturated fatty acid content compared to shortening. Li et al. [20] also presented that the cookie with the replacement of shortening with rice bran wax/soybean oil-oleogel exhibited excellent rheological properties and a sensory evaluation similar to shortening. Especially, cookies are known to contain high fat and high sugar products in bakery food models. For the development of healthier cookies, research is needed to develop a fat substitute using the oleogel rich in unsaturated fat and to explore the most suitable gelators for cookies.

Therefore, in this study, to explore the application of *Tenebrio molitor* larvae oil (TM oil) for food, crude TM oil was extracted, and fatty acid composition and antioxidant activity were investigated. Then, the oleogels were prepared with TM oil and gelators (three types of waxes), and the thermal and rheological properties and their applications as a shortening replacer in a cookie were characterized.

## 2. Results and Discussion

### 2.1. Fatty Acid Composition of Tenebrio Molitor Oil

*Tenebrio molitor* oil (TM oil) was extracted by low-temperature compression to safely apply it to food without using an organic solvent, and its fatty acid composition is presented in Table 1. In a preliminary study, the fat content of *Tenebrio molitor* larvae was found to be 33.3% based on dry matter (data not shown). The saturated fatty acid content of TM oil was 23.8% and their highest percentage corresponded to palmitic acid (16:0, 17.3%). The unsaturated fatty acids content of TM oil was 73.6%, oleic acid (18:1, 46.1%) was the most abundant fatty acid, and linoleic acid (18:2, 25.1%) was the second most abundant unsaturated fatty acid. This result is in agreement with those of previous studies [21,22]. Generally, insect lipids have a high content of unsaturated fatty acids, including linoleic and oleic acids [23]. Because humans cannot synthesize essential fatty acids, such as alpha-linolenic or linoleic acid, the consumption of unsaturated fatty acids such as those found in TM oil is associated with a healthy life. Although TM oil is obtained from animal sources, it was found that the content of unsaturated fatty acid was excellent when compared with the recent research of plant-source oil [24] and microorganism-produced oil [25].

### 2.2. Antioxidant Activities of Tenebrio Molitor Oil

**Figure 1:**
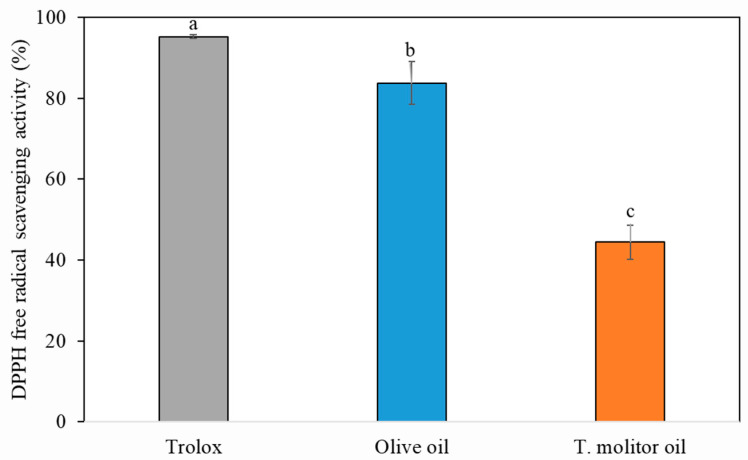
Antioxidant activities of Trolox, olive oil, and *Tenebrio molitor* oil (means with different letters on bars indicate significant differences at *p* < 0.05).

The potential antioxidant activity of TM oil was confirmed by measuring its DPPH radical-scavenging activity. The antioxidant capacities of TM oil were compared with those of Trolox standard and olive oil, which has a similar fatty acid composition. As shown in Figure 1, the DPPH radical-scavenging activity of TM oil was 48.72 *±* 0.60%, although the antioxidant activity of TM oil was lower than that of olive oil. Matia et al. [26] reported that the lipophilic extract of TM was below 0.04 TEAC (mmol TE/100 g) of antioxidant activity, which was also lower than that of olive oil. According to del Hierro et al. [27], a 57% DPPH inhibition was observed in TM extract, and the extraction method of TM might affect the antioxidant activity. The oxidative stability of oleogel manufactured using oil with a high content of unsaturated fatty acid is a very important factor to expand the use of oleogel in the food industry. Recently, to improve the shelf life and nutritional value, the bioactive compounds such as beta-carotene [28], vitamin C [29], proanthocyanidin [30], Co Q10 [31], and curcumin [32] were incorporated with an oleogelator and it has been reported that the oxidative stability of oleogel using them increased. In addition, the development of a new oleogel using oil possessing antioxidant activity could be a more effective strategy to expand the food industrial application.

### 2.3. Thermal Properties of Oleogels

**Figure 2:**
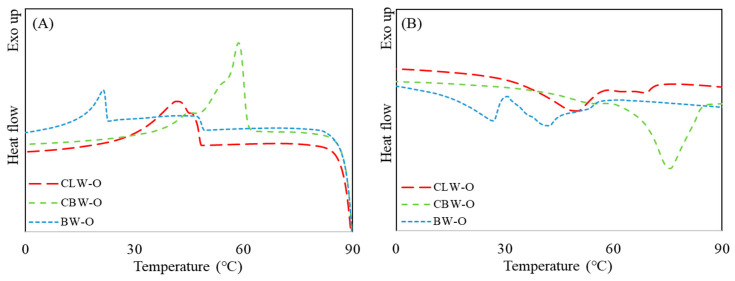
Heating (**A**) and cooling (**B**) thermal properties of oleogels with different wax types by DSC (CLW-O: candelilla wax-oleogel, CBW-O: carnauba wax oleogel, BW-O: beeswax oleogel).

To utilize TM oil as a solid-fat replacer, the TM oil was restructured by oleogelation using different oleogelators (wax types). As clearly illustrated in Figure 6, the TM oil in the liquid form was transformed into the solid structure of the oleogels without chemical changes. As the thermal properties of oleogels are considered to be of important quality in solid fat, the thermal profiles of oleogels with different wax types were investigated and are presented in Figure 2. Depending on the wax type, the oleogels exhibited different crystallization and melting temperatures. In the crystallization thermogram, the peak temperature of BW-O was lowest (25.3 °C), followed by CLW-O (41.4 °C) and CBW-O (58.6 °C). Two separate peaks were observed at approximately 20–50 °C in the melting thermogram of BW-O, indicating the heterogeneity of oleogel formation. On the other hand, the single melting peaks that occurred ranged from 32 to 50 °C and from 60 to 80 °C for CLW-O and CBW-O, respectively. The crystallization (or melting) enthalpy of CBW-O was considerably higher than that of the oleogels, indicating that CBW-O has a higher resistance to temperature changes. Lim et al. [33] prepared canola oil-oleogels by applying similar wax types and found that a higher energy was needed to melt the carnauba wax-oleogel, compared to the canelilla-wax oleogel. Rocha et al. [34] also reported that the melting temperature of candelilla wax/soybean oil (4%) oleogel was 42.1 °C. Compared to thermal properties of this study, it was observed that the thermal characteristics of the oleogel were more affected by the oleogelator (wax type) than by the type of oil. According to Ögütcü [35], the melting temperature of shortening was 58.4 °C, which was the closest to that of CBW-O (58.6 °C). These thermal patterns of the oleogel samples were also explained by their rheological changes with temperature.

### 2.4. Viscoelasticity of Oleogels

**Figure 3:**
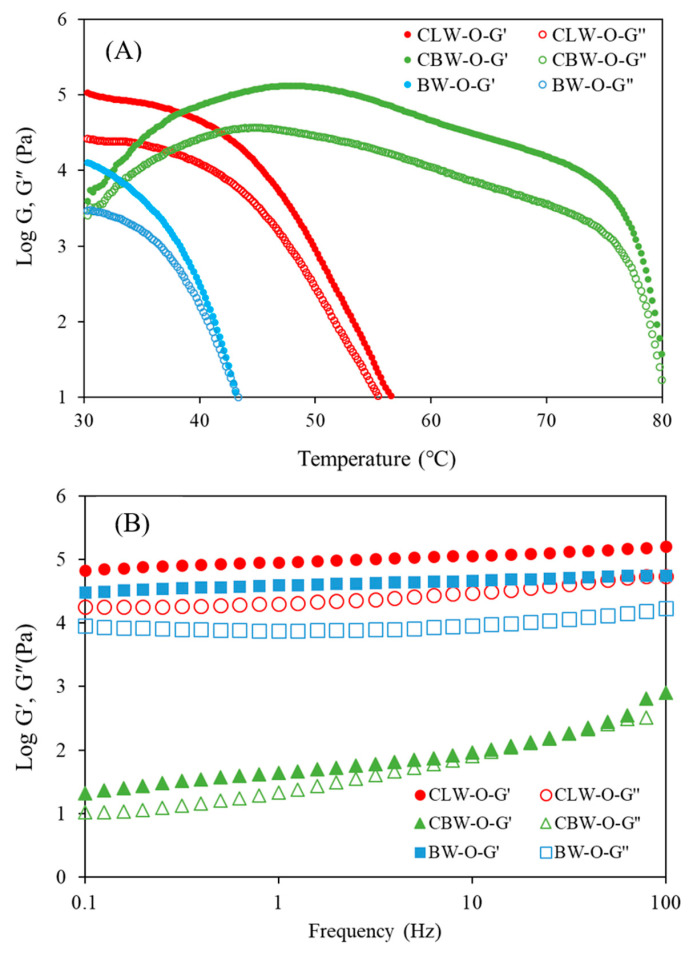
Viscoelasticity of oleogels as a function of temperature (**A**) and frequency (**B**). CLW-O: candelilla wax-oleogel, CBW-O: carnauba wax oleogel, BW-O: beeswax oleogel.

Figure 3A shows nonisothermal rheological properties of oleogels with different wax types, depending on the temperature (50 to 90 °C). For all temperature ranges, the storage modulus (G′ value) was larger than the loss modulus (G″ value), which is associated with a strong three-dimensional network formed by crystal aggregation. The G′ values of CLW-O and BW-O dramatically decreased from 30 °C as the temperature increased, but the G′ of CBW-O increased slightly at up to 50 °C and then decreased above 50 °C. CBW-O exhibited a distinctly higher viscoelasticity, especially at temperatures above 50 °C. These temperatures of slope change were similar to the melting temperatures observed by DSC, showing that first peak observed for thermal analysis is related to breakage of the oleogel network. The rheological properties were affected by melting temperature. In the study of Li et al. [20], the G’ of shortening was decreased slightly at about 45 °C and sharply decreased until 54 °C, which indicated the melting temperature. As indicated by the DSC result, the viscoelastic pattern of shortening was similar to the curve of CBW-O. During the baking process, the change in rheological property of oleogel depending on temperature might be related to the texture and physical properties of the final product.

In addition, the dynamic viscoelastic properties of the oleogels were evaluated as a function of the frequency. Viscoelasticity changes due to the frequency were also different for each oleogel. As shown in Figure 3B, the storage and loss moduli tended to increase with frequency, showing frequency dependence. CLW-O showed the highest viscoelasticity at all frequencies at 25 °C. CLW-O and CBW-O exhibited higher values of G′ than G″, indicating a predominance of elastic characteristics. However, BWO showed a G′–G″ crossover above 10 Hz, which could also be used as a simple criterion for the gel point.

### 2.5. Gel Strength and Oil Binding Capacities of Oleogels

The gel strength of the oleogel samples was investigated by measuring the hardness, which is the force required to cause deformation of the material. As shown in Table 2, the highest hardness values were observed for CLW-O, followed by BW-O and CBW-O. Thus, the TM oil appeared to form a stronger crystalline microstructure derived from candelilla wax. This tendency is consistent with results of Hwang et al. [36], showing that the oleogel with candelilla wax had the highest firmness. Compared to the study of Kupiec et al. [37], the gel strength of oleogel with CLW/rapeseed oil (5%, *w/w*) also showed the highest gel strength, which had a higher value than CLW/TM oil oleogel did. On the other hand, the lowest value was observed in BW/rapeseed oil oleogel, which had a lower value than BW/TM oil oleogel did in this study. Laredo et al. [38] suggested that the hardness of oleogel was related to the fatty acid unsaturation of oil used, and the more double bonds in oil made them have a more tightly aggregated structure.

The oil binding capacity was investigated to evaluate the durability of TM oil binding by oleogelators and the value was calculated by a formula. The highest oil binding capacity was observed in CLW-O (94.18%) and followed by BW-O (93.18%) and CBW-O (92.94) (Table 2). This result presented the relatively high stability of the oleogel structure, which was made with a 10% addition of structure-forming oleogelator in TM oil. The oleogel with CBW had the largest leakage of the oil phase after centrifugation. According to the result of Blake et al. [39], oil binding capacity was related to the crystal size as well as the spatial distribution of these crystals. In addition, Li et al. [40] reported that the oil binding capacity was highly correlated with the G’ values, and the strong networks with high elasticity improved the oil binding capacity. Therefore, the G′ value at 25 °C of CLW-O was highest and it had the lowest oil loss in this study.

### 2.6. Oxidative Stability

**Figure 4:**
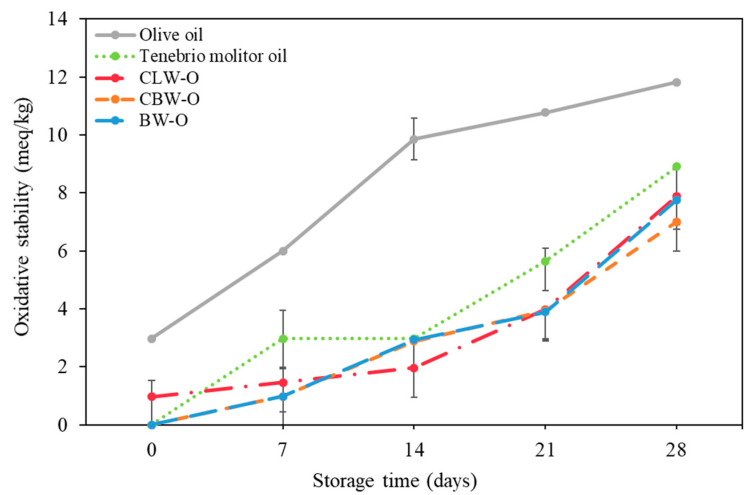
Oxidative stability of olive oil, *Tenebrio molitor* oil, and oleogels.

The oxidative stability of olive oil, TM oil, and oleogels was evaluated by monitoring the peroxide values for 28 days under accelerated conditions (Figure 4), as the peroxide value is considered a measure of the oxidative stability of oil [41]. The peroxide values of all the oil samples and oleogels tended to increase with storage time. After storage for 28 days, olive oil had the highest peroxide value, followed by TM oil and oleogel. This result showed that the restructuring technology of liquid TM oil converted into a solid oleogel was effective in retarding oxidation. This result appears to be consistent with the results of Lim et al. [33], who reported a greater oxidative resistance of solid oleogels with a harder texture. Therefore, the oleogels might be kinetically stable due to their solid-like structure, which may reduce the rate of oxidative reactions. Previous studies showed that oleogelation without additional antioxidants had satisfactory oxidative stability. Luo et al. [42] reported that camellia oil-based oleogel with different oleogelators (2.5% tea polyphenol-palmitate particles and 3.5% citrus pectin) increased the oxidative stability. Oh et al. [43] fabricated the HPMC/canola oil-based oleogel, which decreased the peroxide value by a factor of 1.7 compared to the canola oil.

### 2.7. Color of Oleogel Cookies

**Figure 5:**
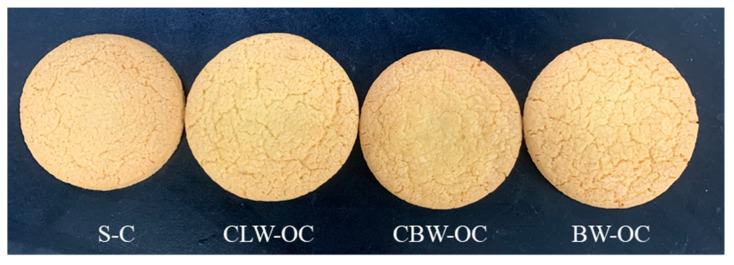
Visual appearance of cookies containing shortening and different oleogels. S-C: shortening cookie, CLW-OC: candelilla wax-oleogel cookie, CBW-OC: carnauba wax-oleogel cookie, BW-OC: beeswax-oleogel cookie.

The effects of replacing shortening with oleogels on cookie quality were characterized in terms of color, geometry, and textural properties. The visual appearance of the cookies made with oleogel is presented in Figure 5; the cross-sectional area increased as the shortening was replaced with oleogel. Table 3 shows that the shortening-cookie (S-C) had the highest value for a* (redness) and b* (yellowness). When the shortening in cookies was replaced with oleogel, a lower a* (redness) value was observed, except CBW-OC. There was no significant difference in the samples in L* (lightness) values. Although the same TM oil was used in all oleogel preparations, some color differences (b* values) occurred in the cookie surfaces because of the different oleogelators used to prepare the oleogels. As can also be seen in Table 3, the effect of shortening replacement with oleogels was investigated on hue angle (H°) and chroma (C*), which is a measure of color nuance and purity of color, respectively. A distinct difference in C* values was detected within the samples, although from the visual appearance of cookies, the difference (*p* < 0.05) in color was hardly observed (Figure 5). The lowest C* was observed in BW-OC. Hue angle did not show a significant difference among the samples without CLW-OC. This phenomenon indicated that the color of wax used in oleogel formulation affected the color of the cookies.

### 2.8. Physical Properties of Oleogel Cookies

As shown in Table 3, the shortening cookie (S-C) had the lowest diameter and highest height, and replacement shortening with oleogel resulted in a cookie with a larger diameter and lower height. The diameter of the cookies prepared with BW-O was higher than that of the cookies made with other oleogels. This result is consistent with that obtained by Yılmaz and Öğütcü [19], who investigated the effect of sunflower wax and beeswax/hazelnut oil-oleogel replacement on cookie quality. The spread factor has been used as a measure of cookie-quality properties [44] and it was indicated that the higher the spread factor, the lower the aeration in the cookie dough. The spread factor for cookies made with shortening was lowest (8.06) compared to those made with CLW-O and BW-O (9.20 and 9.55). According to several previous studies, the replacement of shortening in cookies results in an increased spread factor [13,19]. However, the spread factor of CBW-OC was not significantly different from that of the S-C sample, and it can be concluded that aeration was similar in cookies prepared with shortening and CBW-O due to the similarity of the melting temperature and the curve of the G’ value as a function of temperature. Consequently, the replacement of shortening with CBW-O positively contributed to the physical properties of cookies. Moreover, as shown in the study of Zhao et al. [45], it can be concluded that the hardness of oleogel is not directly correlated to the hardness of cookies.

### 2.9. Texture Characteristics of Oleogel Cookies

The effect of oleogels on cookie quality was investigated by the snapping force, which induces breaks under an applied force, using a three-point bending test (Table 3). The snapping force was significantly decreased by substituting shortening with CLW-O and BW-O. Jang et al. [13] reported that the highest snapping force was for a cookie using shortening, and decreased values for oleogel with candelilla wax were observed. As described earlier, CBW-O had a texture profile most similar to that of S-C, compared to other oleogels. This study suggests that the hardness of cookies can be explained more in connection with the viscoelastic properties of fat at high temperatures rather than the strength of the fat (or oleogel). For the practical application of TM oil, the preparation of oleogel seems to produce a solid fat substitute with nutritional attributes. Moreover, cookies with oleogels containing TM oil and carnauba wax may provide an acceptable method to introduce desirable qualities to consumers. The previous study (2015) with cookies applied by sunflower wax and bees wax/hazelnut oil-based oleogel revealed that the rheological and textural properties of these cookies were not different to the control shortening cookie [19]. On the other hand, the studies of Kim et al. [46] and Oh et al. [18] reported that the saturated fatty acid was reduced by replacing the shortening with the wax-based oleogels. According to these literature studies, saturated fatty acid was 58~74% in shortening and 13~17% in oleogel samples. The replacement of shortening with TM oil-oleogel containing high unsaturated fatty acid (73.6%) can also predict the health effect of reducing saturated fatty acids as reported in the related literature. As the first application of TM oil/wax- based oleogels to foods, the primary research focus of this study was placed on the evaluation of the processing performance of TM oil/wax-oleogels in baked goods as a new solid fat replacer. In a further study, sensory evaluation will be necessary to determine consumer acceptance for the practical applications of TM oil/wax-oleogels.

## 3. Conclusions

*Tenebrio molitor* oil (TM oil), which was extracted at low temperature by compression for utilization in food products without chemical treatment, had a highly unsaturated fatty acid composition, and its antioxidant activity was identified in this study. To use TM oil as a solid fat replacer, an oleogel was successfully prepared using TM oil and three types of natural wax (candelilla, carnauba, and bees), and oleogelation effectively improved the oxidative stability during storage time under the accelerated condition. The hardness values of the oleogels differed depending on the type of oleogelator used. In the case of oleogel with carnauba wax, the highest viscoelasticity above 40 °C was observed and the melting temperature was observed at 75 °C by DSC analysis. As a result of applying the oleogel to cookies instead of shortening, the hardness of oleogel cookies with candelilla wax and beeswax was lower than that of shortening cookies and tended to become softer. In addition, the oleogel cookie with carnauba wax did not show significant differences in the spread factor and texture properties compared to the cookie with shortening, indicating the possibility of serving as an alternative to shortening. The use of TM oil instead of highly saturated fat presents possible opportunities to formulate healthier solid-like goods by improving their fatty acid profiles. Further studies on oleogels containing TM oil in various food models are necessary for their practical solid fat replacement applications.

## 4. Materials and Methods

### 4.1. Oil Extraction from Tenebrio Molitor Larvae

**Figure 6:**
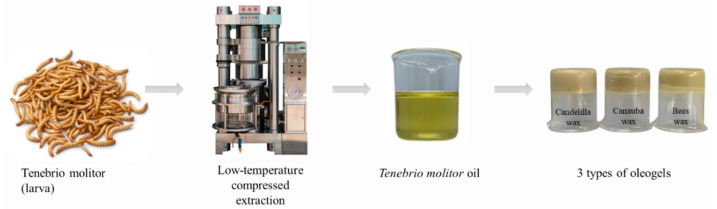
Extraction of *Tenebrio molitor* oil and preparation of oleogels with different oleogelators.

*Tenebrio molitor* larvae were purchased from Myungpyum Co., Ltd. (Seoul, Korea) (Figure 6). (Jangseong-gun, Korea). *Tenebrio molitor* larvae, which were 11–13 weeks old, were fasted for 48 h to empty their gut and dried by microwave. The crude oil from *Tenebrio molitor* larvae (whole shape, not powder form) was extracted with a compressor (Poongjin, Korea) at a low temperature (25 °C) in order to produce it economically through green processing without using organic solvents. The low-temperature extraction method is commonly used for extraction of sesame oil in Korea and was first applied to *Tenebrio molitor* larvae in this paper. After taking 35 kg of *Tenebrio molitor* larvae, the extraction was performed under 700 kgf/cm^2^ of pressure for 25 min, resulting in a 31.8% extraction yield of total weight.

### 4.2. Analysis of Fatty Acid Composition

The fatty acid composition of the Tenebrio molitor oil (TM oil) was analyzed using a gas chromatograph (Hewlett-Packard 6890, Agilent Technologies, Palo Alto, CA, USA) with a flame ionization detector and a SP-2560 column (100 m × 0.25 mm ID, 0.20 mm film) from Supelco (Bellefonte, PA, USA). An internal standard (triundecanoin (C11:0)) in isooctane was added to the TM oil at a concentration of 1000 μg/mL, and fatty acids were derivatized to fatty acid methyl esters (FAME) using BF3/MeOH (14 g/100 g of boron trifluoride). The oven temperature was initially 100 °C, held for 4 min, and then ramped up to 225 °C and held for 20 min. Inlet and detector temperatures were maintained at 225 °C and 285 °C, respectively. The split ratio was 1:200, and the flow rate of the helium carrier gas was 0.75 mL/min.

### 4.3. Antioxidant Activity Measurement

The DPPH radical-scavenging activity of TM oil was evaluated according to the method described by Brand-Williams et al. [47], with some modifications. Briefly, 2 mL of 0.2 mM solution of DPPH (Sigma-Aldrich, St. Louis, MO, USA) was mixed with 2 mL of the diluted TM oil and then incubated in a dark room for 30 min. After incubation, the absorbance of the sample was measured at 517 nm using a spectrophotometer (C40; Implen, München, Germany). Trolox (Sigma-Aldrich) and olive oil (Sajodaerim Co., Seoul, Korea) were used for comparison with TM oil. All measurements were conducted in triplicate, and the DPPH free radical-scavenging activity was calculated using the following formula:

(1) $$\mathrm{DPPH}\;\mathrm{radical}\;\mathrm{scavenging}\;\mathrm{activity}=\frac{A\;\mathrm{Control}\;517\mathrm{nm}-A\;\mathrm{Sample}\;517\mathrm{nm}}{A\;\mathrm{Control}\;517\mathrm{nm}\;}\times 100$$

### 4.4. Preparation of Oleogels

Candelilla wax (Kahl GmbH and Co., KG, Trittau, Germany), carnauba wax (Starlight Co., Fortaleza, Brazil), and beeswax (Hooper Pharm GmbH Co., Hamburg, Germany) were obtained from commercial sources. Oleogels were prepared according to the method of Lim et al. [33]. To prepare the oleogel, the extracted TM oil (90 g) in a glass beaker was placed in an 85 °C water bath for 15 min and mixed with three different oleogelators (10 g): candelilla wax, carnauba wax, and beeswax, until the wax was completely dissolved. The samples were named as oleogelator types: oleogel using candelilla wax (CLW-O), oleogel using carnauba wax (CBW-O), and oleogel using beeswax (BW-O). The concentration of the oleogelator was determined to be 10%, which confirmed the solid-like properties of the oleogel in the preliminary test. After mixing the oleogelator, each oleogel was cooled at room temperature for 30 min and then stored in a refrigerator (5 °C).

### 4.5. Thermal Properties of Oleogels

The thermal behavior of the oleogel samples was analyzed using differential scanning calorimetry (DSC Q200/TGA Q50, TA Instruments, New Castle, DE, USA). The oleogel samples were weighed (10 ± 1 mg) and hermetically sealed in a standard aluminum pan (Tzero Aluminum, TA Instruments, New Castle, DE, USA). An empty pan was used as a reference, and the flow rate of nitrogen gas was 50 mL/min. The oleogel samples were heated from 25 °C to 80 °C at a constant rate of 10 °C/min and held for 5 min. Cooling and heating cycles were then performed. The samples were cooled to −80 °C and then heated to 80 °C at a constant rate of 10 °C/min. The TA Instruments Universal Analysis 2000 software was used to plot and analyze the DSC data.

### 4.6. Dynamic Viscoelastic Properties of Oleogels

The dynamic viscoelastic properties of the oleogels with TM oil were investigated as a function of temperature and frequency. All measurements were conducted using a controlled stress rheometer (AR1500EX, TA Instruments, New Castle, DE, USA) equipped with temperature regulators. The oleogel sample was loaded onto a Peltier plate, and a 40 mm crosshatched probe was compressed with a 1 mm gap. First, the dynamic oscillatory shear storage (G′) and loss (G″) moduli were measured by increasing the temperature from 30 to 80 °C at a heating rate of 5 °C/min. The frequency used in this study was 1 Hz, and the strain (0.1%) was chosen to be within the linear viscoelastic range determined by a strain sweep test. Additionally, a frequency sweep test was applied at 25 °C in the frequency range of 0.1 to 10 Hz under 0.1% strain.

### 4.7. Gel Strength and Oil Binding Capacity (OBC) of Oleogels

The hardness of the oleogels prepared with different wax types was determined using a texture analyzer (CR100, Sun Scientific Co., Ltd., Setagayaku, Japan) equipped with a 20 N load cell. A flat plate probe (5 mm diameter, P/5 cylindrical probe) was used for the puncture experiments at a constant test speed of 60 mm/min, and the penetration distance was 15 mm. The maximum peak force was measured as the firmness from the force–time curve.

The oil binding capacity (OBC) of oleogel samples was measured based on the method described by Yi et al. [48] with some modifications. Briefly, oleogel (1 g) was first weighed (a) and placed into 15 mL conical tubes. The tubes with samples were weighed (b) and centrifuged at 3500 rpm for 15 min. After centrifugation, the oils were removed at the surface, and the sample was weighed (C). The OBC was calculated with the following formulas:

$$\mathrm{Released}\;\mathrm{oil}\;\left(\%\right)=\frac{\left(b-a\right)-\left(c-a\right)}{\left(b-a\right)}\times 100$$

OBC (%)=100−Released oil (%)

### 4.8. Measurement of Peroxide Values

Assessments of the oxidative stability of oleogels with TM oil were conducted by measuring the peroxide value during storage under accelerated conditions (60 °C). The peroxide values of olive oil, Tenebrio molitor oil, and oleogels were compared, and the method of evaluating peroxide values was based on the AOAC official method [49]. Briefly, the sample (1 g) was dissolved in a solution of acetic acid and chloroform (3:2, *v/v*). After 1 mL of potassium iodide solution was added, the sample was allowed to react in the dark for 10 min. Then, 30 mL of distilled water and 1 mL of starch solution (1%) were added, and the sample was titrated with 0.01 N sodium thiosulfate. The peroxide value was expressed as milliequivalents of active oxygen per kg of oil (meqO_2_/kg).

### 4.9. Preparation of Cookies Replacing Shortening with Oleogels

Cookie samples were prepared according to the AACC-approved method (Method 10–52, AACC [50] with slight modifications). For cream production, sugar (252 g), baking powder (4.2 g), skim milk powder (12.6 g), and shortening (126 g) were mixed with a KitchenAid Mixer (Joseph, MI, USA) at a speed of 2 for 2 min. After scraping, the mixture was continuously mixed at a speed of 4 for 1 min. The oleogel replaced shortening 100%, and the cookie samples were named CLW-OC, CBW-OC, and BW-OC, according to the type of oleogel. In a mixing bowl, 112.8 g of cream was placed, and 0.95 M NaHCO_3_ solution, 1.52 M NaCl solution (6 g), water (8.1 g), and wheat flour (120 g) were added and mixed using a KitchenAid Mixer on speed 2 for 3 min. The cookie dough was rolled to a thickness of 10 mm and cut using a 60 mm diameter cookie cutter. After baking cookies in a conventional oven at 205 °C for 10 min, and cooling them down for 60 min, the cookies were sealed in a plastic bag for further analysis.

### 4.10. Color, Geometry, and Texture Measurement of Cookies

The color of the cookies was determined using a colorimeter (CR400; Minolta Co., Kyoto, Japan). Colors are expressed in terms of L* (lightness/darkness), a* (redness/greenness), and b* (yellowness/blueness). Hue angle (H°) and chroma (C*) were calculated based on the following equations [51]:

(2) $$C^{*}=\sqrt{{\left(a^{*}\right)}^{2}+{\left(b^{*}\right)}^{2}\;}$$

(3) $$H{}^{\circ}=tan-1\frac{b^{*}}{a^{*}}$$

The thickness and diameter of the cookies were measured with calipers according to the AACC International Approved Method 10–54.01 (2010). The geometry was measured by rotating the cookies at 90° intervals four times, and the spread factor of the cookies was measured from the ratio of the diameter and thickness of the cookies:

(4) $$\mathrm{Spread}\;\mathrm{factor}\;=\;\frac{\mathrm{average}\;\mathrm{value}\;\mathrm{of}\;\mathrm{diameter}}{\mathrm{average}\;\mathrm{value}\;\mathrm{of}\;\mathrm{thickness}}$$

The textural properties of the cookies were measured using a texture analyzer (CR100, Sun Scientific Co., Ltd., Setagayaku, Japan). The snapping force on the cookies was measured using a three-point bending test. A blade-type probe (0.5 cm thickness and 3 cm width) was used at a test speed of 60 mm/min and a penetration distance of 6 mm.

### 4.11. Statistical Analysis

All experimental results were statistically analyzed using SPSS software (IBM SPSS Statistics 26, IBM Corp., Armonk, NY, USA). Duncan’s multiple range test was performed to determine significant differences among the samples (*p* < 0.05).

## Figures and Tables

**Table 1:** Fatty acid composition of *Tenebrio molitor* oil.

Fatty Acids	% of Total Fatty Acid
Lauric acid (c12:0)	0.3 ± 0.0
Myristic acid (c14:0)	3.6 ± 0.0
Palmitic acid (c16:0)	17.3 ± 1.2
Stearic acid (c18:0)	2.5 ± 0.2
Arachidic acid (c20:0)	0.1 ± 0.0
Saturated fatty acid content	23.8 ± 1.3
Palmitoleic acid (c16:1)	2.3 ± 0.0
Oleic acid (c18:1)	46.1 ± 2.1
Linolenic acid (c18:2)	25.1 ± 0.5
Linolenic acid(α) (c18:3)	1.8 ± 0.2
Gondoic acid (c20:1)	0.5 ± 0.0
Eicosadienic acid (c20:2)	0.1 ± 0.0
Unaturated fatty acid content	73.6 ± 3.8

**Table 2:** Texture properties and oil binding capacity of oleogels with different wax types.

	CLW-O ^(^^1)^	CBW-O ^(^^2)^	BW-O ^(^^3)^
Hardness (N)	2.65 ± 0.01 ^a^	1.21 ± 0.07 ^c^	2.20 ± 0.11 ^b^
Oil binding capacity (%)	94.18 ± 0.23 ^a^	92.94 ± 0.59 ^b^	93.18 ± 0.83 ^b^

^(1)^ CLW-O: candelilla wax-oleogel, ^(^^2)^ CBW-O: carnauba wax oleogel, ^(3)^ BW-O: beeswax oleogel. Different letters indicate significant difference between samples at *p* < 0.05 in the same row.

**Table 3:** Color, physical properties, and texture characteristics of cookies with oleogels.

		S-C ^(^^1)^	CLW-OC	CBW-OC	BW-OC
Diameter (cm)		10.46 ± 0.17 ^c^	10.69 ± 0.00 ^b^	10.63 ± 0.00 ^b^	10.82 ± 0.03 ^a^
Height (cm)		1.30 ± 0.02 ^a^	1.16 ± 0.01 ^b^	1.31 ± 0.03 ^a^	1.13 ± 0.00 ^c^
Spread factor		8.06 ± 0.16 ^c^	9.20 ± 0.14 ^b^	8.09 ± 0.17 ^c^	9.55 ± 0.12 ^a^
Snapping force (N)		80.57 ± 2.99 ^a^	68.01 ± 3.59 ^b^	80.30 ± 2.32 ^a^	58.77 ± 2.28 ^c^
Hunter’s value	L*	59.20 ± 1.79 ^a^	61.49 ± 1.76 ^a^	61.22 ± 1.99 ^a^	61.95 ± 1.94 ^a^
	a*	11.58 ± 0.50 ^a^	10.30 ± 0.80 ^b^	10.69 ± 0.74 ^ab^	10.39 ± 0.65 ^b^
	b*	33.34 ± 0.74 ^a^	32.95 ± 0.57 ^a^	30.62 ± 0.49 ^b^	29.07 ± 0.70 ^c^
Chroma (C*)		35.29 ± 0.54 ^a^	34.53 ± 0.39 ^b^	32.44 ± 0.29 ^c^	30.87 ± 0.45 ^d^
Hue angel (H°)		70.83 ± 1.16 ^b^	73.91 ± 0.24 ^a^	70.75 ± 1.48 ^b^	70.32 ± 1.56 ^b^

^(1)^ S-C: shortening cookie, CLW-OC: candelilla wax-oleogel cookie, CBW-OC: carnauba wax-oleogel cookie, BW-OC: beeswax-oleogel cookie. Different letters indicate significant difference between samples at *p* < 0.05 in the same row.

## Data Availability

The datasets generated for this study are available on request to the corresponding author.
